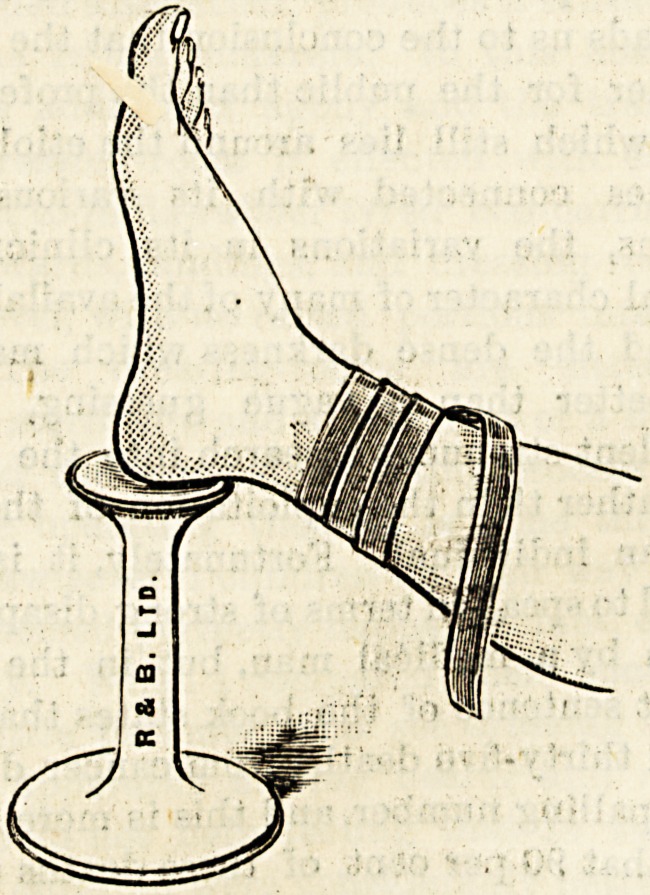# New Appliances and Things Medical

**Published:** 1903-05-02

**Authors:** 


### NEW APPLIANCES AND THINGS MEDICAL.

[We shall be glad to receive at our Office, 28 & 29 Southampton Street, Strand, London, W.C., from the manufacturers, specimens of all new preparations and appliances which may be brought out from time to time.]

### PLASMON ARROWROOT.

(INTERNATIONAL PLASMON, LIMITED, 66 FARRINGDON STREET, LONDON, E.C.)

FROM a dietetic standpoint Plasmon Arrowroot has distinct advantages over the ordinary varieties of arrowroot. As we have frequently stated in these columns, the simple farinaceous foods, though highly valued by the public, are not, as a matter of fact, physiologically suited for the feeding of the sick. It is true that farinaceous foods are easily digested, that they are valuable energy producers, and that they constitute an economical diet for those who perform heavy manual labour; but on the other hand for invalids and those of sedentary habits who require but little fuel for the production of physical labour, they are, at any rate except in very small quantities, quite unnecessary and even harmful. The invalid and convalescent require food for the repair of tissue, and proteid foods are the articles of diet which can perform this task satisfactorily. Plasmon, which contains the proteids of milk, when added to arrowroot, supplies the latter with the tissue-forming elements which it lacks. We therefore regard this new preparation as one of great value for invalid use. It makes a gruel which is not only superior in taste but of genuine nourishing properties.

### THE "SISTER JANE" HEEL SUPPORT.

(REYNOLDS AND BRANSON, LIMITED, BRIGGATE, LEEDS)

THIS is a simple hygienic modification of the ordinary wooden heel support generally used in hospitals for elevating the foot off the ground while dressing a wound of the lower extremity or while applying a bandage. The "Sister Jane" support is of the shape of the old-fashioned wooden stethoscope, with a small concavity at one end for the reception of the heel. It is made of glazed earthenware, and hence can be readily cleaned. It is to be hoped that one or more of these sanitary heel rests will be found in all hospitals and public surgeries.

### DR. HÖGYES' HYGIENIC ASBESTOS SOLES.

As we are receiving inquiries for the above, we wish to state that the address where they may be obtained is Dr. F. Högyes, 18 Queen Victoria Street, E.C. The address was unfortunately omitted in our notice last week.

## Figures and Tables

**Figure f1:**